# Gains of Chromosome 1p and 15q are Associated with Poor Survival After Cytoreductive Surgery and HIPEC for Treating Colorectal Peritoneal Metastases

**DOI:** 10.1245/s10434-019-07923-6

**Published:** 2019-10-16

**Authors:** Malin Enblad, Wilhelm Graf, Alexei Terman, Pascal Pucholt, Björn Viklund, Anders Isaksson, Helgi Birgisson

**Affiliations:** 1 Department of Surgical Sciences, Colorectal Surgery, Uppsala University, Uppsala, Sweden; 2 Department of Immunology, Genetics and Pathology, Experimental Pathology, Uppsala University, Uppsala, Sweden; 3 Department of Medical Sciences, Science for Life Laboratory, Uppsala University, Uppsala, Sweden

## Abstract

**Purpose:**

Genetic alterations in colorectal peritoneal metastases (PM) are largely unknown. This study was designed to analyze whole-genome copy number alterations (CNA) in colorectal PM and to identify alterations associated with prognosis after cytoreductive surgery (CRS) and hyperthermic intraperitoneal chemotherapy (HIPEC).

**Methods:**

All patients with PM, originating from a colorectal adenocarcinoma, who were treated with CRS and HIPEC in Uppsala Sweden, between 2004 and 2015, were included (*n* = 114). DNA derived from formalin-fixed paraffin-embedded (FFPE) specimens were analyzed for CNA using molecular inversion probe arrays.

**Results:**

There were extensive but varying degrees of CNA, ranging from minimal CNA to total aneuploidy. In particular, gain of parts of chromosome 1p and major parts of 15q were associated with poor survival. A combination of gains of 1p and 15q was associated with poor survival, also after adjustment for differences in peritoneal cancer index and completeness of cytoreduction score [hazard ratio (HR) 5.96; 95% confidence interval (CI) 2.19–16.18]. These patients had a mean copy number (CN) of 3.19 compared with 2.24 in patients without gains. Complete CN analysis was performed in 53 patients. Analysis was unsuccessful for the remaining patients due to insufficient amounts of DNA and signals caused by interstitial components and normal cells. There was no difference in survival between patients with successful and unsuccessful CN analysis.

**Conclusions:**

This study shows that gains of parts of chromosome 1p and of major parts of chromosome 15q were significantly associated with poor survival after CRS and HIPEC, which could represent future prognostic biomarkers.

**Electronic supplementary material:**

The online version of this article (10.1245/s10434-019-07923-6) contains supplementary material, which is available to authorized users.

Despite improved treatment with cytoreductive surgery (CRS) and hyperthermic intraperitoneal chemotherapy (HIPEC) for colorectal peritoneal metastases (PM), a significant proportion of patients experience rapid disease recurrence and have limited benefit of the treatment. At present, patient selection for CRS and HIPEC is based on absence of haematogenous spread, resectable PM during surgery, peritoneal cancer index (PCI), and the patients ability to withstand major surgery.^1^ Although PCI is a strong prognostic factor, the macroscopic tumor growth judged by the surgeon does not always correlate to microscopic tumor growth, and low PCI does not always imply a favorable prognosis.^2^–^4^

A novel approach in the rapidly progressing field of PM therefore might be to identify prognostic and predictive molecular biomarkers. Little is known about genetic alterations associated with peritoneal dissemination in colorectal cancer.^5^ In colorectal cancer, chromosomal instability leads to frequent deletions and amplifications throughout the genome causing allelic imbalances and copy number alterations (CNA).^6^ Studies on PM and CNA are scarce, but Diep et al.^7^ demonstrated a larger number of CNAs in peritoneal and liver metastases compared with primary tumors. There also were differences concerning which part of the genome that was affected. This suggests that CNA could play an important role in PM, which needs further evaluation. The purpose of this study were to explore genome-wide CNA in colorectal PM and to identify alterations associated with prognosis in patients treated with CRS and HIPEC.

## Materials and Methods

### Patients and Follow-Up

Between January 2004 and December 2015, 612 patients with PM were scheduled for initial CRS and intraperitoneal chemotherapy at Uppsala University Hospital, Sweden. Patients with inoperable disease (*n* = 76), debulking surgery (*n* = 50), no macroscopic tumor (*n* = 8), and patients receiving sequential postoperative intraperitoneal chemotherapy (*n* = 38) were excluded. Patients with low-grade appendiceal mucinous neoplasms (*n* = 118), noncolorectal primary tumors (*n* = 108), lacking neoplastic epithelium in surgical specimens from CRS (*n* = 41), and patients with pseudomyxoma peritonei (*n* = 51) also were also excluded leaving 122 patients with colorectal and appendiceal PM available for analysis.^8^^,^^9^ Appendiceal tumors were excluded after analysis due to different biology of these tumors and few cases. Baseline variables were retrieved from the medical records, and information about death was recorded from the Swedish population registry (last follow-up February 2017). The study was approved by the regional ethics committee of Uppsala County, Sweden (Dnr 2015/396).

### Cytoreductive Surgery and HIPEC

CRS included peritonectomies, combined with omentectomy and removal of disease-affected organs, as previously described.^10^ HIPEC was performed according to the Coliseum method and was administered for 30–90 min depending on the chemotherapeutic agent used.^11^ The patients had to have adequate renal, liver, and hematopoietic function and WHO performance status of ≤ 2 to be accepted for CRS and HIPEC. The PCI (range 1–39) was used to quantify the extent of macroscopic tumor load in the abdominal cavity at the beginning of surgery.^1^ The completeness of cytoreduction score (CCS) was used to assess the amount of remaining tumor after CRS.^1^

### Histopathology and DNA Preparation

Surgical specimens were fixed in 4% buffered formaldehyde and embedded in paraffin, sliced into 3- to 4-µm sections, and stained with haematoxylin and eosin. An experienced gastrointestinal pathologist (A.T.) reviewed the specimens and identified regions of PM with the maximum tumor cell content. DNA was extracted from 10-µm thin sections of these regions using QIAamp^®^ FFPE Tissue Kit (QIAGEN) according to the manufacturer’s recommendations. DNA was quantified using Qubit^®^ dsDNA HS Assay Kit (Thermo Fisher Scientific). Samples with low concentration of DNA were concentrated using MinElute^®^ Reaction Cleanup Kit (50) (QIAGEN).

### OncoScan^®^

The array analysis was performed according to standard protocols for Affymetrix OncoScan^®^ Arrays (Affymetrix OncoScan^®^ FFPE Assay Kit User Guide (P/N 703175 Rev.2), Affymetrix Inc., Santa Clara, CA). Briefly, 80 ng of total genomic double-stranded DNA was incubated for annealing of molecular inversion probes.^12^–^14^ The gaps formed after the annealing-process were filled with dNTPs followed by DNA amplification through two consecutive steps of PCR. The samples were prepared for hybridization onto the OncoScan^®^ Array after digestion with the *Hae*III enzyme. Hybridized probes were bound to streptavidin-phycoerythrin conjugates using GeneChip^®^ Fluidics Station 450 (Affymetrix Inc.), and arrays were scanned using GeneChip^®^ Scanner 3000 7G (Affymetrix Inc.).

### Data Analysis and Statistics

Microarray data were normalized using Affymetrix OncoScan console 1.3 and segmented using BioDiscovery Nexus Copy Number 8.0 with the TuScan algorithm and default settings. Analyses of allele-specific copy numbers (CN) and average ploidy were performed using Tumor Aberration Prediction Suite (TAPS).^15^ TAPS also was used to calculate frequencies of CNA (gain to > 2 copies per cell, loss to < 2 copies per cell) in the population and for short term (≤ 2 years) and long term (≥ 2 years) survivors over the whole genome.

Correlation between CNA status in each segment of 10 Mbp and survival probability was calculated using log-rank test. To correct for multiple testing and difference in sensitivity of the log-rank test for different group sizes, permutation testing with 50,000 replicates was used to determine the distribution of the smallest *p* value when randomly assigning the patients of the study population into groups based on simulated genome segments. This distribution of extreme *p* values was then used to calculate empirical *p* values for actual genomic segments.^16^^,^^17^

Multivariate Cox proportional hazard regression was used to determine the relative contribution to hazard models of gain of prognostically significant segments of chromosome 15, 1, both 1 and 15, mean CN > 2.5, age, gender, preoperative chemotherapy, signet ring and mucinous histopathology, high PCI (≥ 18) or low PCI (< 18), and CCS 0 or 1 and CEA (carcinoembryonic antigen).^18^ Descriptive data were presented as median with interquartile range if not otherwise specified. The Fisher’s exact test was used to compare proportions, and the Mann–Whitney *U* test was used to compare continuous data of two groups. Statistical analyses were performed with R version 3.1.4 (R foundation for Statistical Computing, Vienna, Austria), when not performed with TAPS.

## Results

### Patients and Follow-Up

Of the initial 122 patients, 114 fulfilled the inclusion criteria after review of histopathological examinations. Patients were excluded because of other primary tumor origin (gastric cancer and breast cancer, *n* = 2) or if no neoplastic epithelium were found in the specimens (*n* = 6). CN analysis was successful in 53 patients. Analysis was unsuccessful because of inability to extract sufficient amounts of DNA from the specimens (*n* = 13), signals caused by interstitial components, and noncancer cells or insufficient amounts of DNA for CN analysis (*n* = 48). Patients with unsuccessful analysis were more likely to have appendiceal cancer, synchronous PM, mucinous and signet ring histopathology, male gender, lower PCI, and lower CEA (Supplementary Table 1). There was no difference in overall survival between patients with successful and unsuccessful CN analysis (*p* = 0.676, Supplementary Fig. 1). CN analyses of appendiceal PM were successful in nine of ten cases due to low tumor cell content. The only patient with appendiceal cancer and successful CN analysis was excluded, leaving 52 patients with colorectal PM for final analysis.

### Copy Number Alterations

**Fig. 1:**
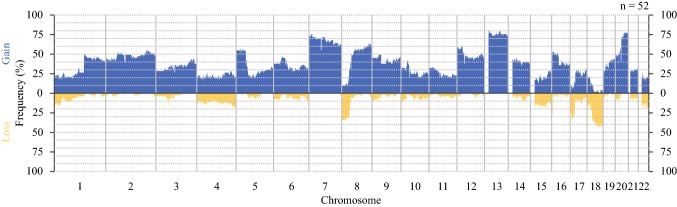
The copy number frequency at each position for all 52 samples. Blue indicates three or more copies. Yellow indicates one or less copies

The frequency of CN gain or loss at each position is shown in Fig. 1. Overall, there was extensive CNA affecting large proportions of the genome. Gain was more common than loss and frequently affected chromosomes 1q, 2p/q, 5p, 7p/q, 8q, 12p/q, 13q, 16p, and 20p/q. Losses were common in chromosome 1p, 4p/q, 8p, 15q, 17p/q, 18p/q, and 22q. Limited tumor heterogeneity was observed in approximately 10% of the samples.

### Prognosis and Copy Number Alterations

**Fig. 2:**
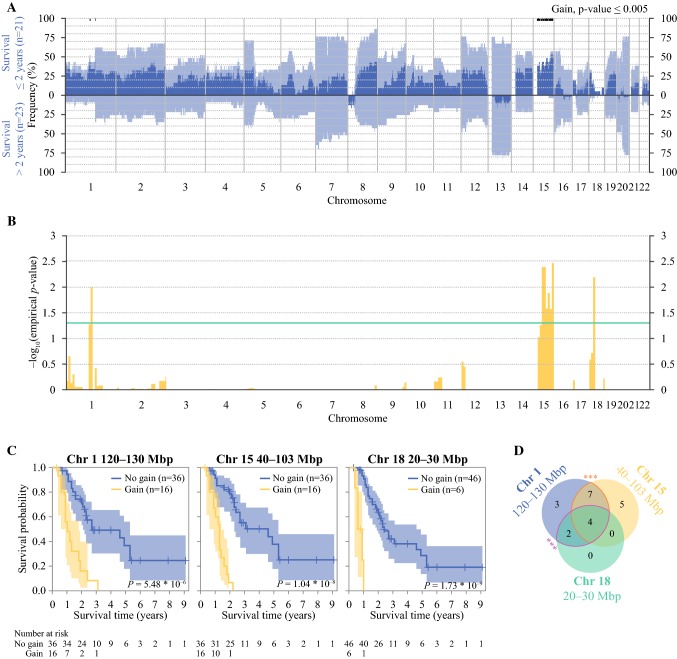
**a** Comparison of copy number frequency of gain between short term survivors (≤ 2 years, top part) and long term survivors (> 2 years, lower part). Light blue is the frequency for each sample and dark blue is the difference. The black bars are regions with *p* ≤ 0.005. **b** Probability of association between gain within segments of 10 Mbp and difference in survival. Values are given as empirical *p* values. Green line is empirical *p* = 0.05. **c** Comparison of survival probability in patients with or without gain within any 10-Mbp segment of chromosome 1 (120–130 Mbp), chromosome 15 (40 and 103 Mbp), and chromosome 18 (20–30 Mbp). **d** Number of samples with gain in any of the focal regions or in a combination of them. There was a significant association between gains of chromosome 15 and 1 (*p* = 0.002) as well as between gains of chromosome 1 and 18 (*p* = 0.0004). *Chr* chromosome

When the population was divided into short-term (≤ 2 years) and long-term survivors (> 2 years) after CRS and HIPEC and compared with respect to frequency of CN, short-term survivors had an overall higher frequency of gains. Gain of parts of chromosome 1p and a majority of chromosome 15q were associated with short-term survival (Fig. 2a, *p* ≤ 0.005). There were no significant losses associated with survival (Supplementary Fig. 2).

In an alternative approach, the population was separated by CN status in all 10-Mbp segments of the genome and analyzed with log-rank test. After correcting for multiple comparisons and using empirical *p* values, gains of parts of chromosome 1p (region 120–130 Mbp) and 18p (region 20–30 Mbp) and gain of major parts of chromosome 15q (region 40–103 Mbp) was associated with shorter survival (Fig. 2b, empirical *p* value < 0.05). The survival probability in patients with and without gain of these regions on chromosome 1p, 15q, and 18p are illustrated in Fig. 2c. Gain of chromosome 18p was found only in patients that also had gain of 1p (Fig. 2d). In addition, a significant association between gains of both 1p and 15q (*p* = 0.002) and of both 1p and 18p (*p* = 0.0004) was found. The region of gain of chromosome 1p included the colorectal cancer-associated genes *REG4* and *NOTCH2* and gain of 15q included the genes *MAP2K1*, *SMAD3*, *SMAD6*, and *IGF1*R, among others. All patients with gain of 1p and 15q had colon cancer and tended to have higher PCI and more often signet ring and mucinous histopathology (Table 1).

**Table 1:** Baseline characteristics of patients with colorectal peritoneal metastases and successful copy number analysis (*n* = 52), stratified by the presence of gain of chromosome 15, chromosome 1, both chromosome 1 and 15, and no gain

	Gain Chr1 *n* (%)	Gain Chr15 *n* (%)	Gain Chr1&15 *n* (%)	No gain *n* (%)
Total	16 (100)	16 (100)	11 (100)	31 (100)
Gender				
Male	7 (44)	7 (44)	5 (45)	11 (35)
Female	9 (56)	9 (56)	6 (55)	20 (65)
Age (median IQR)	55 (47–65)	56 (46–63)	57 (46–65)	60 (54–67)
Primary tumor				
Right colon	7 (44)	9 (56)	6 (55)	16 (52)
Left colon	9 (56)	7 (44)	5 (45)	10 (32)
Rectum	0 (0)	0 (0)	0 (0)	5 (16)
Diagnosis of PM				
Synchronous^a^	9 (56)	10 (63)	7 (64)	16 (52)
Metachronous	7 (44)	6 (38)	4 (36)	15 (48)
Preop. Chemo.	9 (56)	8 (50)	6 (46)	23 (74)
Histopathology				
Mucinous PM	9 (56)	11 (69)	8 (73)	14 (45)
Signet ring PM	2 (13)	4 (25)	2 (18)	2 (6)
PCI (median IQR)	20 (15–24)	21 (18–24)	21 (18–24)	15 (10–22)
CCS				
CC-0	14 (88)	13 (81)	9 (82)	29 (94)
CC-1	2 (13)	3 (19)	2 (18)	2 (6)
CEA (median IQR)	33 (7–103)	14 (4–67)	39 (8–92)	12 (4–39)
Haematog.met.^b^	3 (19)	2 (13)	1 (9)	3 (7)

Values are number of cases with percentage in parentheses if not otherwise specified

*CCS* completeness of cytoreduction score, *Chr* chromosome, *CN* copy number, *IQR* interquartile range, *PCI* peritoneal cancer index, *PM* peritoneal metastases

^a^Diagnosis of PM within 6 months of primary tumor diagnosis

^b^Diagnosis of haematogenous metastasis before or at time of diagnosis of PM

**Fig. 3:**
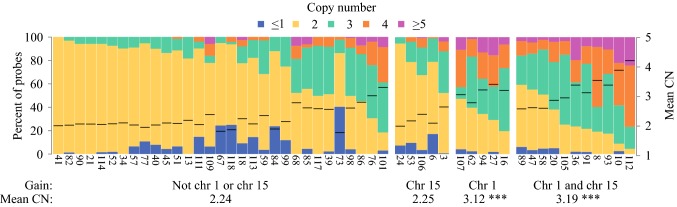
Percent of probes per copy number for all 52 samples separated by samples with gain of chromosome 15, gain of chromosome 1, gains of both chromosome 1 and 15, and no gain of 1 or 15. The copy numbered differed between no gain and gain of chromosome 1 and gains of both chromosome 1 and 15 (*p* = 0.0002 and *p* = 1.91 × 10^−6^ respectively). *Chr* chromosome, *CN* copy number

There was a wide distribution of genomic composition, ranging from minimal CNA (diploid, CN 2) to almost total aneuploidy. Patients with no gain of chromosome 1p or 15q had a mean CN of 2.24. Patients with either gain of chromosome 1p, or both 1p and 15q had a higher mean CN than patients with no gain (Fig. 3, mean CN 3.12, *p* = 0.0002 and mean CN 3.19, *p* = 1.9 × 10^−6^ respectively).

The extensive CNA found in patients with gains of 1p and 15q indicates whole genome duplication.^19^ Survival probability was lower in patients with mean CN > 2.5 (Supplementary Fig. 3, *p *= 0.0372).

In a multivariate hazard model, gain of chromosome 15 and gains on both chromosome 1 and 15 were independently associated with short survival. A mean CN > 2.5 was not an independent prognostic factor. When including clinical variables in the hazard models, the association between poor prognosis and gain of chromosome 15 and gains of both chromosome 1 and 15 remained (*p* = 0.0035 and *p* = 8.0 × 10^−5^). PCI, CCS, and CEA also were associated with poor survival, whereas mucinous histopathology was associated with longer survival (Table 2).

**Table 2:** Multivariate cox proportional regression analysis of predictors of survival in patients with colorectal peritoneal metastases

	Hazard ratio	95% confidence interval	*p* value
Gain			
Chromosome 1, not 15^a^	2.86	0.80–10.2	0.1077
Chromosome 15, not 1^b^	7.42	2.28–24.2	**0.0009**
Chromosome 1 and 15	16.39	5.66–47.5	**2.6 × 10**^**−7**^
Gain and mean CN > 2.5			
Chromosome 1, not 15^a^	3.08	0.68–14.02	0.1460
Chromosome 15, not 1^b^	7.30	2.22–24.07	**0.0011**
Chromosome 1 and 15	17.66	4.59–67.99	**2.9 × 10**^**−7**^
Mean CN > 2.5	0.90	0.29–2.81	0.8569
Chromosome 15			
Gain Chr 15 versus no gain	5.12	1.71–15,32	**0.0035**
Gender	1.22	0.44–3.36	0.7045
Age	1.003	0.96–1.05	0.9057
Preop. Chemo.	1.87	0.64–5.49	0.2537
Mucinous histopathology	0.45	0.15–1.33	0.1480
Signet ring histopathology	1.71	0.45–6.58	0.4326
PCI. ≥18 versus <18	9.12	2.61–31.91	**0.0005**
CCS. CC-1 versus CC-0	8.46	1.97–36.32	**0.0041**
CEA	1.01	1.00–1.01	**0.0116**
Chromosome 1 and 15			
Gain Chr 1 and 15 versus no gain	10.62	3.28–34.36	**8.0** × **10**^**−5**^
Gender	1.80	0.63–5.18	0.2761
Age	1.02	0.97–1.07	0.3894
Preop. Chemo.	3.35	1.05–10.62	**0.0405**
Mucinous histopathology	0.26	0.08–0.84	**0.0242**
Signet ring histopathology	0.76	0.19–3.06	0.6943
PCI. ≥ 18 versus < 18	13.38	3.28–34.36	**5.7** × **10**^**−5**^
CCS. CC-1 versus CC-0	13.64	3.06–60.79	**0.0006**
CEA	1.01	1.00–1.01	**0.0102**

Significant *p* values > 0.05 are given in bold

*CCS* completeness of cytoreduction score, *CN* copy number, *Chr* chromosome, *PCI* peritoneal cancer index

^a^Gain in any 10-Mbp segment of chromosome 1 (120–130 Mbp) and no gain of chromosome 15

^b^Gain in any 10 Mbp segment of chromosome 15 (40–103 Mbp) and no gain of chromosome 1

## Discussion

In this study, colorectal PM had CNA in abundance, and gains of parts of chromosome 1p and major parts of chromosome 15q were independently associated with poor prognosis after CRS and HIPEC. This is the first study to focus on genome-wide CNA in colorectal PM, and a prognostic biomarker may save these patients from unsuccessful surgery and present an opportunity for alternative treatment.

Colorectal PM had a wide range of CNA ranging from diploid to almost total aneuploidy. Genetic alterations in primary colorectal tumors have been thoroughly described by the Cancer Genome Atlas Network and the most frequent CNAs in the PM resembled those found in primary tumors. However, gains were more common and present for almost all chromosomes and pronounced differences were gain of 2p/q, 5p, 12p, and 16p in the PM.^6^ So far, other comparable studies on colorectal PM and CNA are scarce, but in 2004, Diep et al.^7^ analyzed 10 primary carcinomas, 14 local recurrences, 7 PM, and 42 liver metastases using comparative genomic hybridization and observed gains of 5p and 12p more frequently in PM. Kleivi et al.^20^ found increased expression of genes on 5p being more common in PM than in primary tumors and liver metastases. These differences between primary tumors and PM suggests that PM acquire additional genetic alterations and that it is probably not enough to analyze the primary tumor when aiming to predict prognosis or response to therapy. Despite the lack of studies on CNA and PM, there are studies on CNA in relation to events in cancer progression, liver metastases, and overall survival, but the same gain of 1p and 15q has not been described.^21^^,^^22^

This study was designed to identify CNA associated with prognosis after CRS and HIPEC when dividing the studied population into short- and long-term survivors, gains of chromosome 1p and 15q were associated with poor prognosis. To avoid conclusions based on dichotomized survival-groups, the genome was divided into 10-Mbp segments and assessed with log-rank test. After multiple testing correction by permutation testing and using empirical *p* values, gains of 1p and 15q were still associated with poor prognosis and so was gain of chromosome 18p. However, gain of 18p was always combined with gain of chromosome 1p, and interpreted as secondary to alterations of 1p. In addition, gains of 1p and 15q also were combined in the majority of cases, which was independently associated with poor prognosis when analyzed together with well-established prognostic factors, such as PCI and CCS.

It is not clear whether chromosomal instability is an initiating event or a consequence of the malignant transformation process. Therefore, it is difficult to predict the effect on tumor suppressors and promoters. However, chromosome 1p and 15q harbors many colorectal cancer-related genes that could play an important prognostic role. To name a few, the affected parts of chromosome 1 include the genes *REG4* and *NOTCH2*. REG4 is involved in cell regeneration and proliferation and is overexpressed in colorectal cancer and its metastases.^23^ Interestingly, it also works as a promoter of gastric PM.^24^ The Notch pathway is involved in epithelial to mesenchymal transition, which eventually leads to migration of cancer cells.^25^ However, increased *NOTCH2*-expression has been associated with a favorable prognosis.^26^ Almost the entire chromosome 15q was affected, including *MAP2K1, SMAD3, SMAD6*, and *IGF1R*. Dysfunctional SMAD-protein leads to ineffective TGFβ-signaling and tumor growth.^6^^,^^27^ IGF1R is known to be overexpressed in colorectal cancer, and its activation initiates the well-established MAPK (including MAP2K1) and PI3-K pathways in colorectal cancer. Finally, inhibition of IGF1R is one of the targets of upcoming anticancer drugs.^28^

Patients with gains of 1p and 15q had an average CN > 2.5, indicating whole genome duplication, which is a frequent event in colorectal cancer.^19^^,^^29^ Survival probability was found to be decreased for patients with CN > 2.5 but was not an independent prognostic factor. Theoretically, gains of 1p and 15q could be the result of losses in other regions but not in 1p and 15q after whole genome duplication. However, the mechanism behind gains of 1p and 15q remains unknown.

This study has limitations that have to be mentioned. First, < 50% of included samples were successfully analyzed, due to insufficient amounts of DNA and signals caused by interstitial components and normal cells. In addition, only one sample per patient were analyzed, and we were therefore not able to analyze intersample heterogeneity. The method is therefore dependent on tumor composition and not ideal for routine analysis of PM specimens. However, there was no difference in survival between successfully and unsuccessfully analyzed samples, which was relevant, because the goal of the study was to explore genetic alterations and to identify alterations associated with prognosis. That being said, the method is feasible for an exploring analysis, but a future validation is desirable. Second, the risk of multiple comparisons is inevitable with high-resolution genome-wide analyses, but correction was made as described above. Third, the studied population was small and pooled towards aggressive disease when excluding *Pseudomyxoma peritonei*, thus not entirely representative of the heterogeneous group of peritoneal dissemination. However, the studied population represents patients with the greatest need of prognostic and predictive molecular biomarkers.

In conclusion, this study described the extensive but varying CNA in colorectal PM. Gains of parts of chromosome 1p and of major parts of chromosome 15q were significantly associated with poor survival after CRS and HIPEC, which could represent future prognostic molecular biomarkers.

## Electronic supplementary material

Below is the link to the electronic supplementary material.

Comparison of survival probability in patients with successful and unsuccessful copy number analysis (TIFF 74150 kb)

Comparison of copy number frequency of loss between short-term survivors (≤2 years, top part) and long-term survivors (>2 years, lower part). Orange is the frequency for each sample and red is the difference (TIFF 63281 kb)

Comparison of survival probability in patients with mean CN ≤ 2.5 and mean CN > 2.5. *CN* copy number (TIFF 74324 kb)

Supplementary material 4 (DOCX 13 kb)
