# Supplementary figures and images for: Gains of Chromosome 1p and 15q are Associated with Poor Survival After Cytoreductive Surgery and HIPEC for Treating Colorectal Peritoneal Metastases
Source: Ann Surg Oncol. 2019 Oct 16;26(13):4835–42. doi: 10.1245/s10434-019-07923-6 (PMC6863794; doi:10.1245/s10434-019-07923-6)

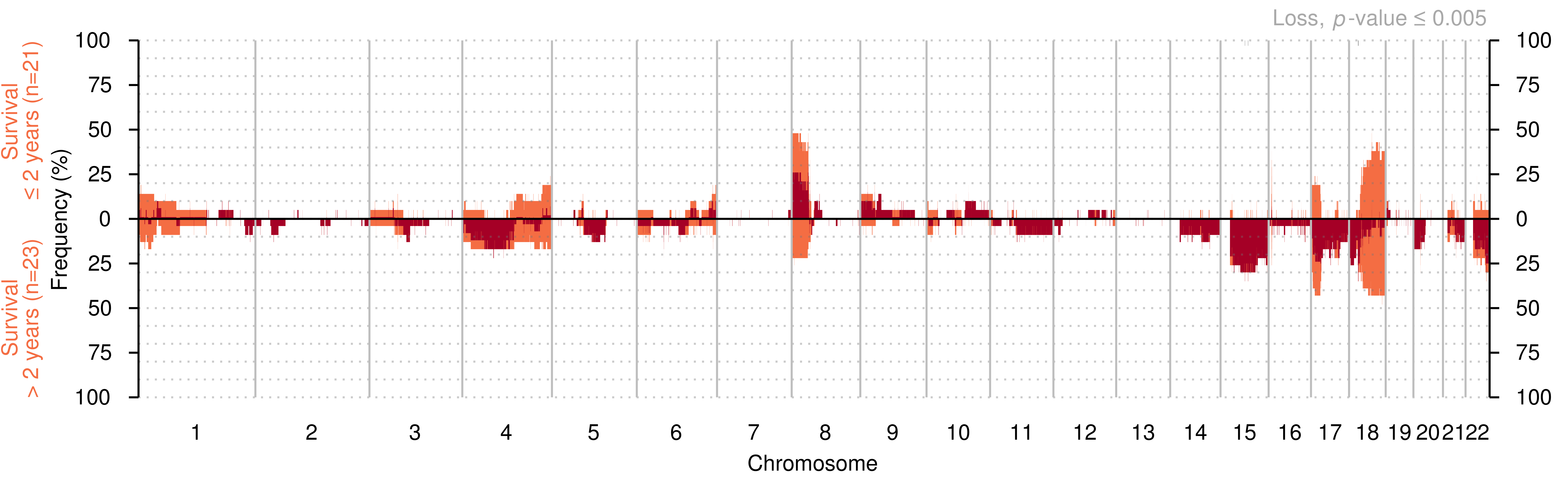

Supplement: Supplementary file 2 — Comparison of copy number frequency of loss between short-term survivors (≤2 years, top part) and long-term survivors (>2 years, lower part). Orange is the frequency for each sample and red is the difference (TIFF 63281 kb) [file 10434_2019_7923_MOESM2_ESM.tiff]
