# Supplementary material for: Gains of Chromosome 1p and 15q are Associated with Poor Survival After Cytoreductive Surgery and HIPEC for Treating Colorectal Peritoneal Metastases
Source: Ann Surg Oncol. 2019 Oct 16;26(13):4835–42. doi: 10.1245/s10434-019-07923-6 (PMC6863794; doi:10.1245/s10434-019-07923-6)
Supplement: Supplementary file 4 — Supplementary material 4 (DOCX 13 kb) [file 10434_2019_7923_MOESM4_ESM.docx]

**Supplementary Table 1**. Baseline characteristics of patients with appendiceal and colorectal peritoneal metastases (n=114), stratified by successful and unsuccessful copy number analysis.

|  | Successful CN-analysis  n (%) | Unsuccessful CN-analysis  n (%) |
| --- | --- | --- |
|  |  |  |
| Total | 53 (100) | 61 (100) |
| Gender |  |  |
| Male | 21 (40) | 34 (56) |
| Female | 32 (60) | 27 (44) |
| Age (median IQR) | 58 (50 − 65) | 56 (46 − 65) |
| Primary tumor |  |  |
| Appendix | 1 (2) | 9 (15) |
| Right colon | 26 (49) | 32 (52) |
| Left colon | 21 (40) | 15 (25) |
| Rectum | 5 (9) | 5 (8) |
| Diagnosis of PM |  |  |
| Synchronous PM^a^ | 29 (55) | 42 (69) |
| Metachronous PM | 24 (45) | 19 (31) |
| Preoperative chemotherapy | 35 (66) | 47 (77) |
| Years since surgery  ( median IQR) | 6 (3 – 8) | 6 (4 – 9) |
| Histopathology |  |  |
| Mucinous PM | 27 (51) | 38 (62) |
| Signetring PM | 7 (13) | 14 (23) |
| PCI (median IQR) | 18 (11 − 24) | 14 (9 − 24) |
| CCS n (%) |  |  |
| CC-0 | 47 (89) | 53 (87) |
| CC-1 | 6 (11) | 4 (7) |
| CC-2 | 0 (0) | 4 (7) |
| CEA (median IQR) | 15 (4 − 45) | 4 (2 − 11) |
| Haematogenous metastasis^b^ | 7 (13) | 4 (7) |

Values are number of cases with percentage in parentheses if not otherwise specified.

^a^Diagnosis of PM within six months of primary tumor diagnosis. ^b^ Diagnosis of haematogenous metastasis prior to or at time of diagnosis of PM. CCS, completeness of cytoreduction score; CN, copy number; IQR, inter quartile range; PCI, peritoneal cancer index; PM, peritoneal metastases
